# White matter microstructure associated with the range of attentional and impulsive performance in school-aged children

**DOI:** 10.1192/j.eurpsy.2022.251

**Published:** 2022-09-01

**Authors:** A. Gagnon, M. Descoteaux, C. Bocti, G. Grenier, V. Gillet, J. Posner, A. Baccarelli, L. Takser

**Affiliations:** 1 University of Sherbrooke, Department Of Pediatrics, Sherbrooke, Canada; 2 University of Sherbrooke, Sherbrooke Connectivity Imaging Laboratory (scil), Sherbrooke, Canada; 3 University of Sherbrooke, Department Of Medecine, Sherbrooke, Canada; 4 CIUSSS de l’Estrie-CHUS, Research Center On Aging, Sherbrooke, Canada; 5 Duke University, Department Of Psychiatry, Durham, United States of America; 6 Columbia University Mailman School of Public Health, Department Of Environmental Health Sciences, New York, United States of America; 7 University of Sherbrooke, Department Of Psychiatry, Sherbrooke, Canada

**Keywords:** Diffusion MRI, Attention/Impulsivity, White matter (WM), Pediatric

## Abstract

**Introduction:**

Inhibition capabilities have been shown to be a strong predictor of social and educational life outcomes (Mischel & Ebbesen, 1970; Shoda et al., 1990). Inhibition capabilities have an enormous impact on attention and impulsivity (Bari & Robbins, 2013). These two executive functions are associated with numerous psychiatric disorders but are not well understood in terms of white matter (WM) connectivity (Puiu et al., 2018). Novel techniques and statistical approaches in neuroimaging bring us closer to a biologically sustained model.

**Objectives:**

This research aims to: 1) identify WM connections associated with attention/impulsivity performance and 2) characterize the differences in WM microstructure associated with the variation of the performance.

**Methods:**

157 children (GESTE cohort, 8-12 years, 27 Dx ADHD, 2 Dx ASD) with b=1500mm^2^/s, 2mm isotropic dMRI acquisitions were included. Tractography was performed with TractoFlow pipeline (Theaud et al., 2020). Dimensionality reduction of diffusion metrics yielded two components : microstructural complexity (DTI Metrics, AFD & NuFo) and axonal density (AFD_fixel) (Chamberland et al., 2019). Attention/impulsivity were evaluated with the CPT3. Multivariate linear regression was performed in python.

**Results:**

Lower microstructural complexity was associated with poorer attentional performance on regions of the parietal lobe to the occipital gyrus (P-O, *p*=0.044, R^2^=0.14, Figure 1.) and the Broadman’s area 8 to area 6 (SF8-SF6, *p*=0.002, R^2^=0.12, Figure 1.). Lower axonal density was associated with a less impulsive pattern on SF8-SF6 (*p*=0.001, R^2^=0.13, Figure 1.). Results remained significant when removing children with an ADHD or ASD diagnosis.

**Figure:**
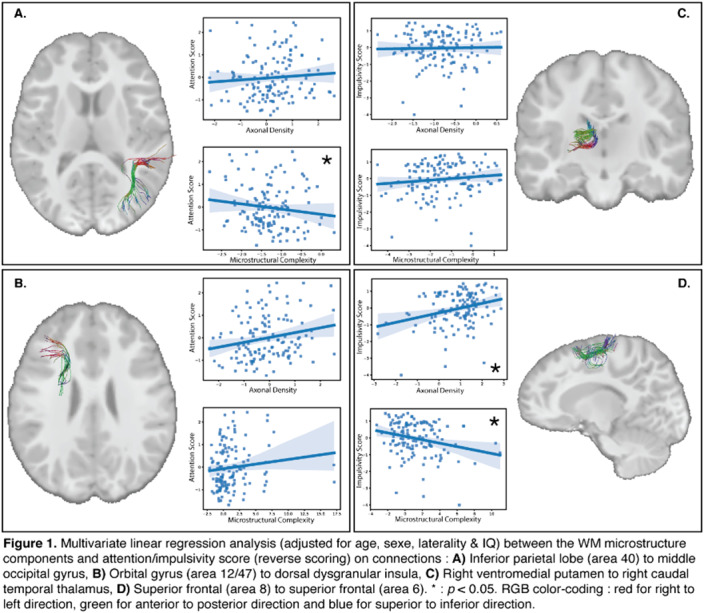

**Conclusions:**

We identified underlying difference in WM microstructure that may be associated with the variation in attention/impulsivity performance in school-aged children.

**Disclosure:**

No significant relationships.

